# The miRNA–target interactions: An underestimated intricacy

**DOI:** 10.1093/nar/gkad1142

**Published:** 2023-11-30

**Authors:** Caroline Diener, Andreas Keller, Eckart Meese

**Affiliations:** Saarland University (USAAR), Institute of Human Genetics, 66421 Homburg, Germany; Saarland University (USAAR), Chair for Clinical Bioinformatics, 66123 Saarbrücken, Germany; Helmholtz Institute for Pharmaceutical Research Saarland (HIPS)–Helmholtz Centre for Infection Research (HZI), Saarland University Campus, 66123 Saarbrücken, Germany; Saarland University (USAAR), Institute of Human Genetics, 66421 Homburg, Germany

## Abstract

MicroRNAs (miRNAs) play indispensable roles in posttranscriptional gene regulation. Their cellular regulatory impact is determined not solely by their sheer number, which likely amounts to >2000 individual miRNAs in human, than by the regulatory effectiveness of single miRNAs. Although, one begins to develop an understanding of the complex mechanisms underlying miRNA–target interactions (MTIs), the overall knowledge of MTI functionality is still rather patchy. In this critical review, we summarize key features of mammalian MTIs. We especially highlight latest insights on (i) the dynamic make-up of miRNA binding sites including non-canonical binding sites, (ii) the cooperativity between miRNA binding sites, (iii) the adaptivity of MTIs through sequence modifications, (iv) the bearing of intra-cellular miRNA localization changes and (v) the role of cell type and cell status specific miRNA interaction partners. The MTI biology is discussed against the background of state-of-the-art approaches with particular emphasis on experimental strategies for evaluating miRNA functionality.

## Graphical Abstract

**Graphical Abstract:**
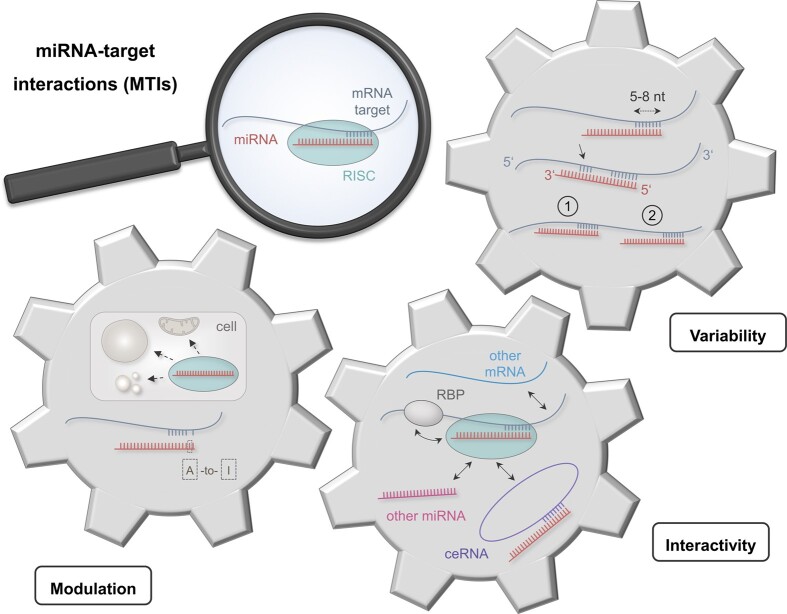

## Introduction

Since their discovery in 1993 (1), microRNAs (miRNAs, miRs) have emerged as potent modulators of cellular gene expression. They are involved in the post-transcriptional regulation of almost all cellular processes and play consequently important roles in the development of many diseases. Accordingly, miRNAs have attracted great interest as potential novel tools for diagnosis and even therapy (2,3). The highly complex nature of the regulatory networks centered around miRNAs has, however, dampened the hopes initially associated with a rapid use of miRNAs in a clinical context. While one is only slowly beginning to acknowledge the complexity of miRNA networks, many supposedly reliable findings must be regarded as preliminary. In addition, it is certainly not least due to the rapid development of miRNA research that many central terms, like the one of a miRNA ‘target’, are far from being uniformly and clearly defined. The lack of generally accepted definition renders statements blurred that operate with such terms. For example, many studies with rather heterogenous definitions of miRNA targets refer to the statement that 60 % of all protein coding genes are potential targets (4). Such ambiguities have serious consequences if falsely defined targets are used in the context of miRNA–target networks. These networks have already an inherent fuzziness about them since they are by no means static structures but are subject to constant dynamic changes, not at least in response to varying cellular conditions (5–7). It is of utmost importance to achieve a greater clarity about the terminology including the definitions of miRNA targets and miRNA–target interactions.

Here, we provide a broad and systematic overview on the complex miRNA–target interactions (MTIs) with a special consideration of state-of-the art approaches to experimentally assess MTIs. To this end we emphasis the factors that contribute most to the great variability of miRNA-to-target binding, the impact of the miRNA localization on their functionality and the mutual stochiometric effects between miRNA and mRNA targets. Finally, we shed light on databases and their capacity to further our understanding of MTIs.

### MiRNA biogenesis and database entries of miRNAs and their targets

**Figure 1.**
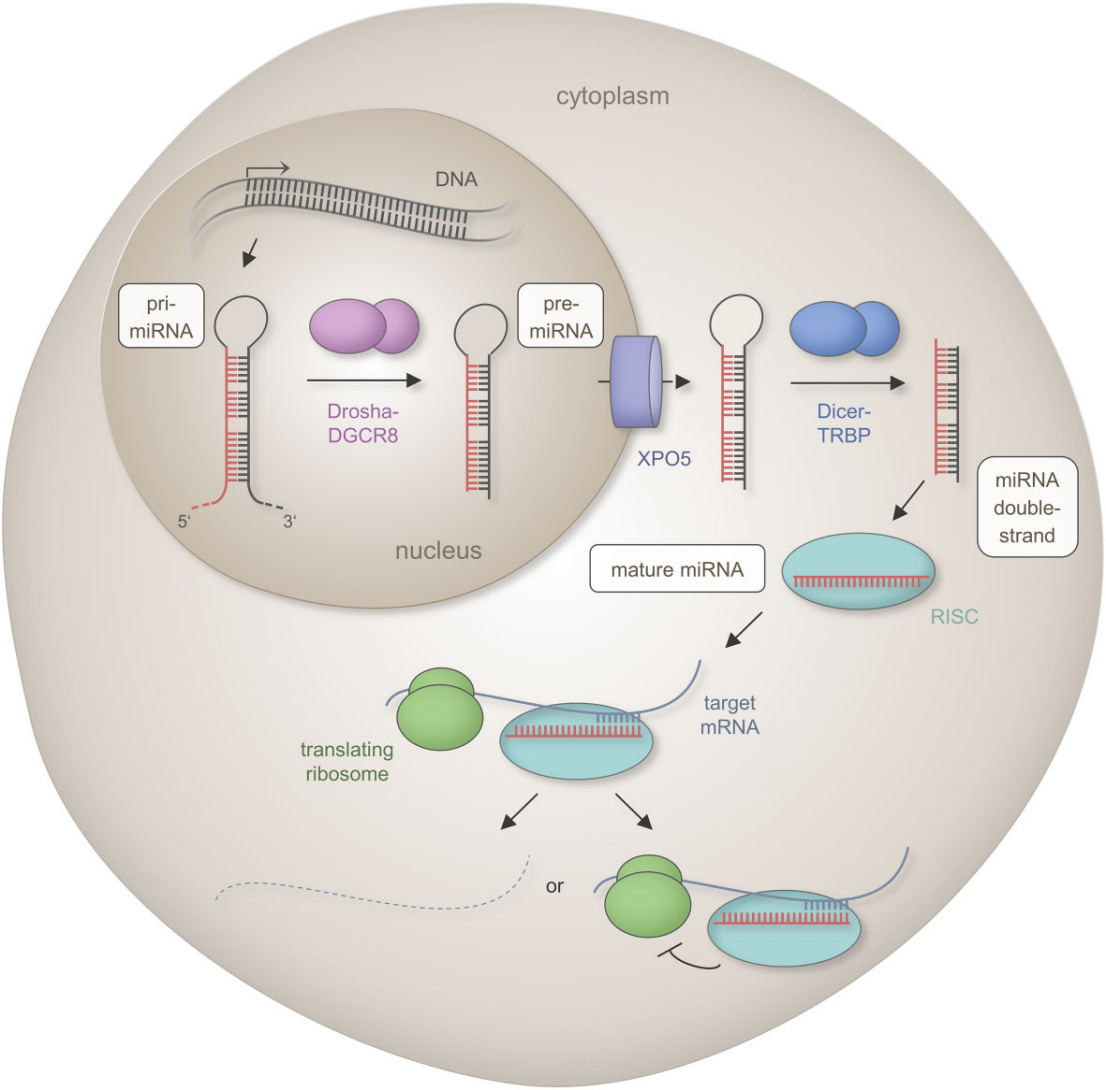
Scheme of the canonical miRNA biogenesis pathway. The first step of the canonical mammalian miRNA biogenesis encompasses the transcription of a primary miRNA (pri-miRNA) from endogenous DNA loci by RNA polymerase II. The primary transcript includes an imperfect hairpin structure that is cleaved by the DROSHA-DGCR8 complex into a 60–90 nt long precursor miRNA (pre-miRNA) with a two nucleotide (nt) 3′ overhang. The pre-miRNA is transported from the nucleus to cytoplasm through the export protein XPO5 in a RAN-GTP dependent manner. The cytoplasmatic miRNA becomes a substrate for the RNase DICER that forms a complex with the double-stranded RNA-binding protein TRBP. Following removal of the loop structure, a miRNA duplex of 19–22 nt in length interacts with proteins of the Argonaute (Ago) family. One strand is incorporated into the RNA induced silencing complex (RISC). The strand with a higher content of purines and a lower thermodynamic stability of the 5′ end takes the dominant biological functionality i.e. it acts as functional miRNA that confers post-transcriptional regulation through RISC catalyzed mRNA degradation, mRNA destabilization or translation repression. [The functional portion of the miRNAs are indicated in red, the mRNA target is shown as a solid blue line, and the degraded mRNA as a dashed blue line. Interactions between miRNA and mRNA are indicated by opposite comb-shaped lines. The Drosha–DGCR8 complex is depicted by pink bodies, the export protein XPO5 by a violet cylinder, the DICER-TRBP complex by blue bodies, the RISC by a turquoise body and ribosomes by green bodies.]

Endogenous miRNAs arise during a complex biogenesis process, of which the main features are summarized in Figure 1. In brief, a hairpin-formed transcript is generated from endogenous DNA loci and stepwise processed into a 19–22 nucleotide (nt) miRNA duplex structure (8,9). Within the cytosol, this mature miRNA interacts with proteins of the Argonaute (Ago) family and is subsequently incorporated into the RNA induced silencing complex (RISC) (10). Canonical miRNA–target interactions are mediated by the ‘seed region’ that covers the nucleotides 2–8 at the miRNA′s 5′-end (11). Reverse-complementary miRNA binding sites, which are also referred to as miRNA responsive elements (MREs), are commonly located within the 3′ Untranslated Regions (3′UTRs) of the targeted messenger RNAs (mRNAs) (11,12), but can also map within 5′UTRs or the protein coding sequence (13,14). The binding ultimately results in a decrease of the corresponding protein levels, due to for example a RISC catalyzed decapping or deadenylation, degradation or inhibition of protein translation (15–17). The miRNA-coupled RISC (miRISC) can interfere with the translation machinery at various stages of the translation process, including initiation, post-initiation and elongation (18). Under specific conditions, as for example during cell cycle arrest, some miRNAs can also promote protein translation (19). This unusual miRNA functionality has first been reported in 2007 but there is a lack of further studies to substantiate this finding (20).

In the early times of miRNA research, single miRNAs were identified by classical Sanger sequencing and their cellular expression status was often experimentally verified (21). But high-throughput technologies have led to a tremendous increase in the amount of miRNA sequence data (22). Sequencing-based miRNA identification resulted in the annotation of numerous miRNAs, which found their way into various databases, such as miRBase (21–23). However, as of today only ∼5 % of the human miRNA entries appear to be experimentally verified by other techniques such as northern blotting (23). Systematic evaluations indicate that many of the annotated sequences likely do not represent true miRNAs, but rather other RNA species or even sequencing artifacts (21,23,24). A premature and wrong annotation as true miRNAs has consequences not only for the understanding of biological networks, whose structure may be judged completely incorrect by considering false miRNAs, but also for clinical settings in which miRNAs and/or their targets are envisaged as starting points for therapeutical interventions (2,25). This caveat is acknowledged in recent miRBase releases by a rating of confidence for miRNA candidates. The rating relies on specific criteria like the presence of characteristic 3′ overhangs as part of the mature miRNA duplex (22). Here, again the dimension of the problems becomes evident. Only ∼35% of the human miRNA entries in the miRBase (v22.1) are classified as ‘high confidence’ miRNAs. Therefore, other databases were developed to complement and extend the miRBase (26). The miRCarta repository aims to provide a sensitive collection of every transcribed non-coding small RNA with properties similar to miRNAs, with the rational to prevent that studies repeatedly claim novel miRNA candidates that have in fact been already reported by others (27). The MirGeneDB has emerged as the best resource for high fidelity resource for metazoan miRNAs. It excels by considering evolutionary aspects on miRNAs and in that all entries are manually curated by consequently applying established hallmarks of miRNA processing (28). In that, MirGeneDB has emerged as a second reference repository for miRNAs in addition to the miRBase.

The complex situation of miRNA identification is reflected in a relative broad range of estimates on the total number of miRNAs. Depending on the selected source of primary data e.g. MirGeneDB (v2.1) or miRBase (v22.1) the listed number of mature human miRNAs varies between 630 and 2 656 (22,28). In *vitro* studies indicate 750–900 human pri-miRNAs as potential DROSHA substrates (29,30). The true number of mature human miRNAs that are experimentally verified seems to be in between these counts and amount to slightly over 2 000 of which not all are annotated in miRbase yet (23). Overall, database entries of miRNAs should be viewed with some caution or better yet skepticism, as long as there is no thorough and robust characterization of a deposited miRNA. As a final remark, even constantly curated and updated databases do not prevent, that formerly annotated miRNAs, which have been removed from databases, are still investigated in recent publications as for example miR-1273g, which despite being withdrawn from the miRBase in version 22 (22) is still analyzed as a functional miRNA in seven publications since 2020. Removal from the database does, however, not necessarily imply the absence of functionality of such miRNAs, especially not the absence of non-canonical functions.

As for databases that list experimentally supported miRNA targets, the DIANA-TarBase allows retrieval of positive and negative miRNA targets per species, methodology, cell type and tissue (31). Verified MTIs from curated articles and CLIP-seq data are collected in the miRTarBase. It includes information on single-nucleotide polymorphisms, disease-related variants related to the binding efficiency of miRNA to their targets and extracellular miRNA expression profiles (32). Despite their usefulness, database entries of miRNAs and the targeted mRNAs should be viewed with some caution or better yet skepticism, as long as there is no thorough and robust characterization of the deposited miRNAs and miRNA targets.

### MiRNA–target binding

#### Seed length, 3′ extended binding, and seedless binding

**Figure 2.**
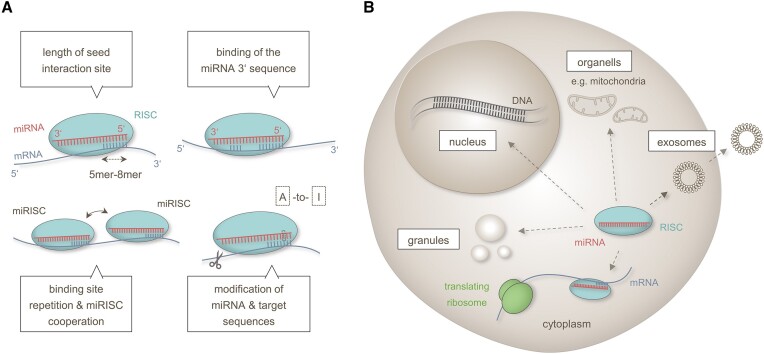
Overview on miRNA–target bindings and miRNA localization changes. (**A**) The regulatory capacity of MTIs is a function of the length of binding interaction between the miRNA seed sequence and the mRNA target, the presence of 3′ binding sites, the cooperation between multiple miRNA-responsive elements and the modification of both, the miRNAs and target sequences through e.g. A-to-I RNA editing or 3′UTR shortening. (**B**) Intra-cellular miRNA sub-localizations include membranous comparts like the nucleus or mitochondria and non-membranous comparts like granules. Extracellular miRNA localization can result from secretion of vesicles like exosomes. [MiRNAs are indicated as red lines, mRNAs as blue lines, interactions between miRNA and mRNA by opposite comb-shaped lines. Length variations are depicted by dashed two-sided arrows and translocations by solid two-sides arrows. Ribosomes are shown as green bodies, RISC as turquoise bodies. Sequence shortening is symbolized by cutting scissors and sequence exchanges by dotted rectangles.]

The efficiency of miRNA–target interactions (MTIs) depends on various factors as detailed in the following and summarized in Figure 2A. The RISC-coupled miRNA interacts with its specific mRNA target by Watson–Crick base-pairing. As abovementioned canonical target binding occurs at the seed region i.e. the nucleotides 2–8 of the miRNA′s 5′ end. As a rule of thumb, there is a hierarchy in the regulatory efficiency depending on the number of seed nucleotides involved in the target interaction (8mer > 7-mer-m8 > 7-mer-A1 > 6-mer) (33,34). However, even 5-mer MTIs could play essential roles for the miRNA targeting as recently shown for miR-34a-5p in human cells (35). Beyond the seed region, additional binding of the miRNA′s 3′ sequence, in the following referred to as 3′ extended binding, can also affect the target regulation. Various studies have shown that human miRNAs of the same family e.g. the let-7 family, can regulate different sets of targets, even though these miRNAs share the same seed region and similar 5′ sequences (5,14). It has been shown as early as in 2007 that extended base paring within the 3′ region of a miRNA can enhance its regulatory efficiency on certain targets. This binding is commonly denoted as ‘supplementary binding’ (36). In addition, 3′ extended binding can also compensate for mismatches within the miRNA seed region. The latter one is denoted as ‘compensatory binding’ (4). While former analyses assumed a principal involvement of the miRNA 3′ nucleotides 13–16 (36), recent data provide evidence that 3′ extended binding can encompass the entire 3′ miRNA sequence (37). The manifestation of 3′ extended binding modes appears to depend on various factors, such as the distribution of G residues within the miRNA sequence (37).

In rare cases, MTIs have been described to even take place without the involvement of the seed region (38). This ‘seedless’ pairing mode is likely prompted by a weak pairing stability of the seed region, due to an AU-rich sequence composition (39) and can be supported by an interplay with nearby canonical miRNA binding sites as shown by analyses in *Caenorhabditis elegans* (*C. elegans*) (40). It remains to be seen if these results are generalizable and if they are applicable to human cells.

#### Repetitive binding and associated binding sites

There is accumulating evidence that the number and distribution of miRNA binding sites within the 3′UTR likely impacts the regulation of specific targets (41). Repetitive binding sites for the same miRNA and adjacent binding sites for different miRNAs can both result in a higher regulatory efficiency (42,43). In general, a distinction must be made between functional cooperativity and binding cooperativity. While functional cooperativity describes the summation of miRNA-related effects, binding cooperativity is characterized by the physical interaction of multiple RISCs, which support each other in their binding to the respective mRNA target (43). In the broadest sense, functional cooperativity can not only be the targeting of multiple miRNA responsive elements within the same mRNA but also of different mRNAs within the same functional pathway (43,44). In the latter case, the summative effects lead to efficient regulation of the affected pathway. As for the binding cooperativity, it has long been assumed that it requires a close localization of the interacting binding sites at distances between 8–39 nt (36,45). These distance restrictions that rely on a linear perspective have been challenged by recent studies that suggest a further impact of the target secondary structure (46). Overall, the interactivity of binding sites renders the analysis of MTIs rather complicated, since the effect of a single miRNAs can only be adequately described in the context with co-expressed miRNAs, with each of the resulting MTIs depending on factors like the accessible seed sequence and the target structure.

#### Editing within and outside of the miRNA seed region

Adding a further level of complexity, miRNAs as well as mRNA target sequences are not static but are subject to changes. Sequence changes through RNA editing can e.g. be introduced into the miRNA seed region thereby altering its binding capacity to target mRNAs (47). Likewise, RNA editing events that occur outside the seed region can impact the processing and the loading efficiency of miRNAs into the RISC, thereby contributing to a flexible responsiveness to different cellular conditions (48). Editing events have been identified at various stages of the miRNA maturation process (48,49) and miRNA editing has been described for many different human tissues, particularly for neuronal cells (49). Corresponding sequence changes are attributed to ADARs (adenosine deaminases acting on RNAs) causing exchanges of adenosine (A) to inosine (I). Additionally, cytidine deaminase enzymes (AID/APOBECs family) have been described to cause cytidine (C) to uridine (U) editing. Particularly, the A-to-I miRNA editing has frequently been shown to alter the targeting of oncogenes in context with cancers (50). There is recent evidence for a preferential editing of distinct miRNA subpopulations that share specific precursor secondary structures i.e. efficient double-strand pairing at the farthest opposite end (‘root region’) of the hairpin-loop (47). In contrast, the C-to-U miRNA editing is much less studied and apparently occurs only under specific physiological conditions such as cellular hypoxia (51). Although not the immediate focus of this review, the role of different miRNA isoforms (isomiRs) that becomes increasingly more recognized (52–55) needs to be acknowledged to ultimately understand the relationship between altered miRNA levels and their regulatory effects.

#### Editing and length variations of target 3′UTRs

Besides its bearing for microRNA sequences, RNA editing events can also affect the 3′UTR sequence of the mRNA targets to vary e.g. the accessibility of certain binding sites (56). As a further factor, the length of the 3′UTRs can also be subject to changes (57). As recently shown, the prevalence of long 3′UTR variants with multiple miRNA binding sites likely affects the miRNA mediated target regulation in mammalian axon growth (41). The 3′UTR length variations can also affect the formation of RNA secondary and tertiary structures, which in turn can change the accessibility of the miRNA binding sites (58,59). Extensive 3′UTR shortening as the result of alternative transcript processing has for example been described to cause a peripheral positioning of miRNA binding sites, thereby enhancing the miRNA binding capacity during the proliferation of human embryonic fibroblasts (7).

### Functional effects of different miRNA localization

Besides their known cytoplasmic functions in the translation process, miRNAs can also exert other specific regulatory tasks depending on their subcellular localization (Figure 2B). The intra-cellular localizations include membranous organelles like the nucleus or the mitochondria and non-membranous compartments as for example ribonucleoprotein granules (60).

There is accumulating evidence that miRNAs, which are localized within the nucleus are involved in the regulation of the transcription process (61–64). To exert miRNA guided transcriptional regulations, the mature miRNA-coupled RISC is transported from the cytoplasm into the nucleus. Nuclear miRNA shuttling is likely mediated by specific transporter proteins including importin-8, importin-α/β and XPO1 (65–67). Since the amount of a translocated miRNA very likely impacts its regulatory function in the nucleus, it is expected to be controlled by a number of different factors including distinct sequence motifs (6,68,69). In addition, specific environmental conditions such as hypoxic stress have been associated with miRNA shuttling between the cytoplasm and the nucleus (6). Within the nucleus, the miRISC complexes can bind to promoter sequences and to DNA regulatory elements, thereby impacting the recruiting and binding of transcription factors and chromatin remodeling factors (70,71). The miRNA guided expressional regulations have been described to result either in transcriptional gene activation (TGA) or transcriptional gene silencing (TGS) (71). Examples include the transcriptional upregulation of *STAT3* by miR-551b-3p (72) or the downregulation of the transcription factor EB by miR-30b-5p (73).

As for their intracytoplasmic sub-localization, it has been hypothesized that miRNAs such as miR-762 can be transported into the mitochondria to silence mitochondria resident transcripts (74). A dysregulation of this miRNA has recently been associated with immunological changes in Parkinson′s Disease and with myocardial infarction (74,75). There is, however, an ongoing debate on the mechanism of mitochondrial microRNA (mitomiR) translocation. Recent analyses indicate that shuttling into the mitochondria may be mediated by interactions of the miRNA coupled RISC with specific transport proteins such as the Polynucleotide Phosphorylase (PNPase) (76). As of now the circumstantial evidence for a presumed miRNA functionality in mitochondria awaits further experimental confirmation (77).

Among the miRNAs that co-locate with non-membranous granules, there are miRNAs that group together with processing bodies (P-bodies/PBs) (78). Recent analyses found both, active and inactive miRNAs enriched in PBs. While regulatory active miRNAs were found to only temporarily associate with the P-bodies, inactive miRNAs were found to be stably anchored, suggesting a mechanism, which sets unused miRNAs apart (79).

In addition to their localization within cellular compartments, miRNAs are also found in vesicular structures like exosomes. It is generally assumed that these miRNAs can be transferred between distant cells and likely act as part of the intercellular communication (80). However, the specific steps of the corresponding transport processes are only partially understood. Most recent findings suggest that vesicular miRNA export is mediated by specific RNA-binding proteins like Alyref and Fus (81). A selective sorting of mRNAs into exosomes appears to be directed by cell type specific consensus sequences of 4–7 nt (EXOmotifs), while an intracellular retention of miRNAs is mediated by different motifs (CELLmotifs) (81).

### Mutual stochiometric effects between miRNAs and target genes

#### Stochiometric effects of miRNA on mRNA targets

**Figure 3.**
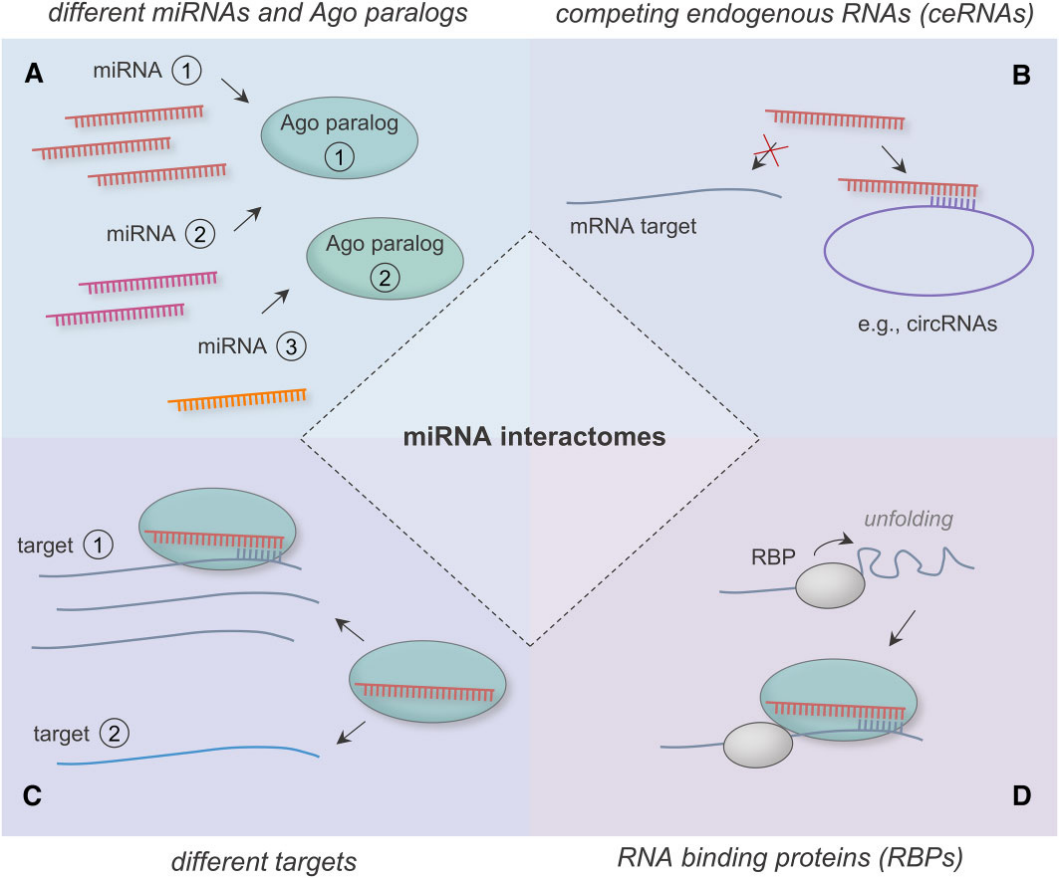
Scheme representation on the diversity of miRNA interactomes. (**A**) Different miRNAs compete for cellular resources, including the available RISCs. There are specific preferences of the miRNAs for their interaction with different Ago paralogs as central part of the RISC. (**B**) Target mRNAs and competing endogenous RNA species, like circular RNAs (circRNAs), contest for miRNA binding. (**C**) Different mRNAs compete for RISC bound miRNAs. (**D**) RNA-binding proteins (RBPs) modulate accessibility of a mRNA target site for miRNAs. [Different miRNAs are depicted in red, pink and orange, respectively. Different mRNAs (targets) are shown in shades of blue and ceRNAs in violet. A different molecular abundance is indicated by a different number of miRNAs or mRNAs, respectively. RISCs are shown by turquoise and RNA binding proteins by greyish bodies. Functional interactions are indicated by arrows and sequence interactions by opposite comb-shaped lines.]

The miRNA function is further modulated by interactions between different miRNAs, mRNA targets, other types of non-coding RNAs and RNA binding proteins (Figure 3). The stochiometric ratios of these interacting partners play a critical role for the regulatory effects of miRNAs. Various studies have shown that altered miRNA expression levels impact the miRNA targeting suggesting a concentration dependence of miRNA regulatory effects (5,82). A meaningful cellular miRNA effect is assumed to require a minimal cellular miRNA abundance (5,83). For miRNAs that are present in sufficient quantities their biological effect is likely determined by a competition for limited cellular resources, most notably the available RISCs (84,85). Quantitative studies describe cellular miRNAs levels in the range of 10–10^5^ miRNA molecules per cell (86), with only a small fraction of highly abundant miRNAs (86,87). The small fraction of these highly abundant miRNAs most likely exert dominant regulatory effects in these competitions (83). However, the role of the numerous non-highly abundant miRNAs should not be underestimated. These miRNAs have also the potential to exert important regulatory functions, for example through a high binding affinity to cellular key targets like transcription factors (88,89). In addition, non-highly abundant miRNAs may potentiate their regulatory effectiveness not only by functional cooperation, but also by multiple rounds of target regulation (90–93).

The miRNA functionality is furthermore influenced by the four Ago protein paralogs (Ago1-4) that are expressed in mammalian somatic cells. Although, there are some redundancies between the miRNA related function of these paralogs (94), there are specific functional differences including their ability to efficiently cleave a targeted mRNA (95). Different tissues and cell types show variable expression levels of the different Agos (96), possibly contributing to context specific miRNA functions. Individual miRNAs and even different isomiRs have been found to preferentially interact with specific Ago paralogs (97–99). Furthermore, the expression of specific Ago subtypes can be impacted by the prevalence and abundancy of certain microRNAs, suggesting a feed-back mechanism (100).

#### Stochiometric effects of mRNA targets on miRNAs

The miRNA expression not only impacts the target expression but vice versa the amount of mRNA target impacts the miRNA abundance by affecting the turnover rate of a miRNA. Analyses of mammalian cells provide evidence that 3′ extended and seedless binding modes can trigger decay of miRNAs through a process termed ‘target-directed miRNA degradation’ (TDMD) (101,102). Current findings also show that the prevalence of certain mRNA targets and a high target-to-miRNA ratio likely promote TDMD, indicating a multifactorial and context dependent coordination of the process (103,104). As for the underlying mechanism, it is assumed that the Ago changes its conformation during the target interaction, so that the miRNA′s 3′ end is exposed and becomes accessible for 3′-tail trimming and exonuclease-based degradation (105). Another quantitative aspect is, that a high target-to-miRNA ratio increases the likelihood for specific miRNA target interactions at the expense of other target bindings (88,89). This target competition is ultimately the result of a molecular titration process (89,106).

#### Impact of miRNA sponges and RNA-binding proteins

A final but relevant quantitative aspect of MTIs concerns the embedding of miRNA–target networks in larger networks of various competing endogenous RNAs (ceRNAs), also referred to as endogenous miRNA sponges. Due to competing binding sites, these ceRNAs likely keep miRNAs from binding to their mRNA targets (107,108). The ceRNAs include various types of non-coding RNAs, such as long non-coding RNAs (lncRNAs) or circular RNAs (circRNAs) (107,109–111). Mathematical modeling predicts favorable settings for miRNA–ceRNA interactions under the condition that nearly equimolar expression rates are given (108,112). Beyond the interactions of miRNAs with their mRNA targets and other types of RNAs, there is accumulating evidence, that the prevalence of RNA-binding proteins (RBPs) can also impact miRNA-mediated target regulations (113). Although most RBPs likely modulate local 3′UTR secondary structures of mRNA targets to enhance miRNA binding capability (114), there are also reports about antagonistic effects of RBPs, including Human antigen R (HuR), through the blocking of miRNA binding sites (115,116). Additionally, miRNA expression levels can be controlled by direct and indirect effects of RBPs, including AUF1 (AU-binding factor 1), DDX17 (DEAD-box helicase 17) and ILF3 (interleukin enhancer binding factor 3) (117–120).

### Experimental access to MTIs

MiRNA regulations usually result in rather moderate effects on the expression levels of single targets (121). The limitations are compensated in cases where the target proteins of these miRNAs map within the same regulatory pathway (122–124). However, the detection of the effects of a given miRNA on single target mRNAs remains rather challenging. To this end common criteria and tools have been developed for the *in silico* prediction of potential miRNA targets. These *in silico* tools have thoroughly been reviewed elsewhere (125). Functional enrichment analysis by other computational tools like ‘GeneTrail’ (126), can further enhance the validation rates of experimental testing strategies (122,127). In the following, we focus on the experimental validation of MTIs.

#### Induced altered miRNA expression

In recent years, a variety of different strategies have been developed to allow for efficient miRNA manipulations. Common strategies to induce miRNA overexpression include the use of expression plasmids or synthetic miRNAs (miRNA mimics) (2). However, miRNA overexpression approaches usually lead to unphysiologically high expression levels and to off-target effects, in part due to the utilization of cellular resources like RISCs, which will be less or no longer available for endogenous miRNAs (128).

Common strategies for miRNA inhibition include assays to either down-regulate or functionally obstruct endogenous miRNAs. As most recently shown, miRNA down-regulation can for example be achieved by sequence specific degradations using miRNases conjugated to miRNA-binding antisense oligonucleotides (129). Alternatively, a functional inhibition of miRNAs can be achieved by complementary ‘RNA zippers’ that allow for a sequence specific end-to-end connection of endogenous miRNA molecules (130). To abolish miRNA expression, miRNA knockouts have been achieved by genome editing techniques including TALEN (Transcription Activator-like Effector Nuclease) or CRISPR/Cas9 (Clustered Regularly Interspaced Short Palindromic Repeats/CRISPR‐associated protein 9) (131–133). As for the miRNA overexpression, off-target effects have been reported for both miRNA inhibition and miRNA knockouts (128,134). This issue has recently been addressed by sequence specific nucleic acid masks and peptide nucleic acids (PNAs) that were designed to block binding sites on mRNA targets without affecting the miRNAs themselves. These approaches have been shown to effectively inhibit miRNA functions in MTI studies (135,136).

An issue that is highly relevant for both, induced miRNA overexpression and inhibition, concerns the distance between the time-point of manipulation and the time-point of measurement. The detection of related down-stream effects is affected by cellular turnover rates. In detail, the amounts of transiently transfected oligonucleotides for miRNA manipulations are subject to cellular turnover (137–139). The same applies to miRNA targets, which can be measured as mRNAs or proteins, each with their specific cellular half-lives (140,141). Although, there are attempts to provide guidelines for the estimation of optimal time-points for miRNA downstream analysis (142), this will remain a task that has to be optimized, anew in each experiment.

#### MiRNA and target expression analyses

**Figure 4.**
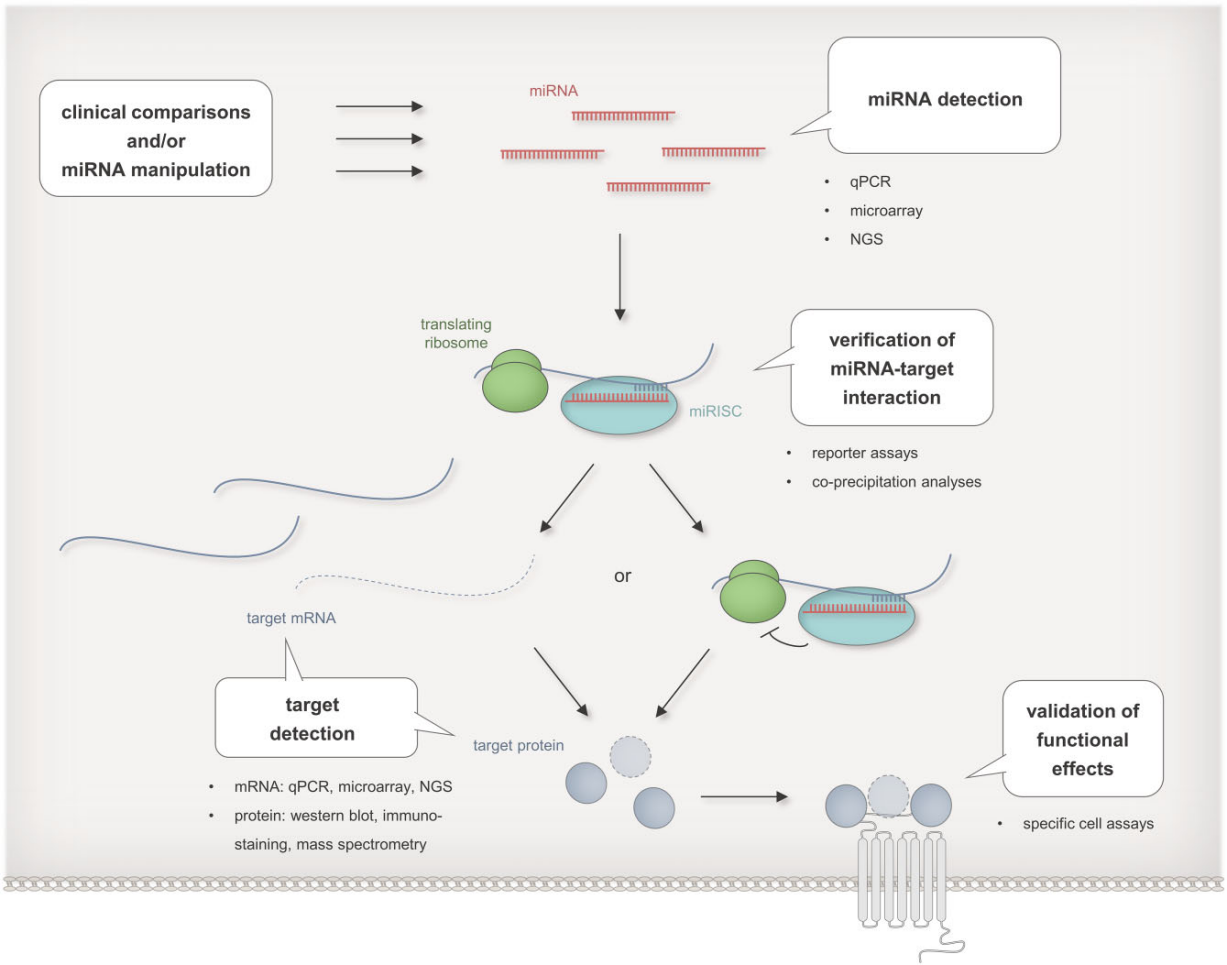
Experimental strategies for the identification of miRNA targets. MiRNA targets can be identified by comparisons of e.g. healthy vs. diseased cells or by downstream analysis upon experimental miRNA manipulations. Differential abundances of miRNAs and target mRNA are detectable by qPCR, microarray analyses, or next generation sequencing (NGS), differential levels of target proteins by western blot, immunostaining or mass spectrometry, direct interaction between miRNAs and their target mRNA by reporter assays or co-precipitation analyses, and down-stream miRNA effects by proliferation assays, viability assays or by assays tailored for specific miRNA regulated cell functions. [MiRNAs are indicated in red, target mRNAs in blue, and target proteins as blue spherical bodies. Interactions between miRNA and mRNA are depicted by opposite comb-shaped lines. Degradation of mRNA is indicated by dashed lines and reduction in protein expression by dashed outlines. Functional effects are symbolized by a grey shaded receptor icon. Ribosomes are shown as green bodies, RISCs as turquoise bodies.]

Various experimental strategies have been employed to examine different aspects of the miRNA regulatory process (Figure 4). First information on the miRNA-mRNA target interplay can be gained by approaches that integrate cellular expression data of miRNAs and their predicted targets (131,143,144). Conclusive experimental data are obtained from comparisons between healthy and diseased cells or as the result of an experimentally altered miRNA expression. Expression analysis on the RNA level is carried out by either low-throughput methods like quantitative PCR (qPCR) or high-throughput RNA detection methods like RNA-sequencing (145) that have largely replaced formerly employed array-based techniques. Target analysis on the mRNA level is frequently complemented or even replaced by protein analyses, although most of the miRNA regulations appear to show their primary effects on the mRNA level (17,146). Depending on the task, proteins are analyzed by either low-throughput methods like Western blotting or immunostaining (147,148) or high-throughput methods like mass spectrometry (149,150).

A major advantage of these descriptive miRNA–target analyses is their independence of specific experimental settings, such as a model cell line. However, the detection of inverse correlation may be simply due to chance, especially when complex miRNA–target data sets are analyzed using a limited number of samples. Unfortunately, there are numerous manuscripts that fall in this category of randomness by reporting inverse miRNA–target correlations in a far too small number of samples. A further limitation of the observative approaches is that their data mostly result from the analysis of a single point in time. As addressed above, this caveat is for example relevant for processes in which target effects of a miRNA are found only with an extended time delay. Here, target effects can be readily overlooked in scenarios where a decay of the miRNA had occurred at the time when its biological effect becomes measurable by an altered target abundance. Likewise, miRNA regulatory effects may only be observable at specific cellular stages or at specific time-points of a disease process. Recent analyses address this challenge by evaluating time-series expression data during biological processes like for example T-cell activation (151). Other approaches attempt to associate miRNA and target expression levels by correlating datasets, even if they stem from different tissues (152,153). These analyses allow to identify common miRNA regulations but fall short in identifying cell type specific miRNA functions.

#### Reporter assays and immunoprecipitation-based approaches

A still limited in number of experimental approaches allow to validate for the miRNA-to-target binding. A common method of choice are reporter assays that use recombinant plasmids, which include a reporter gene (e.g. luciferase or GFP gene) under the post-transcriptional regulatory control of the potential target′s 3′ UTR sequence (154,155). The MTI is determined by measuring the reporter activity upon overexpression or inhibition of the respective miRNA (156). The experiment can be by sequence mutation of the potential miRNA binding site, either by a complete deletion or an exchange of the wildtype MRE, reverting the miRNA′s impact on the reporter (157). One has, however, to bear in mind that alterations of the 3′UTR potentially create alternative binding sites and change the interactivity not only with the designated miRNA, but also with other endogenous miRNAs and RBPs (158,159). MiRNA seed mutations represent another option for the validation of reporter assays of MTIs. Here, one has to bear in mind that artificial sequence changes within the seed region potentially affect the half-life of miRNAs and may alter their targeting of endogenous mRNAs and the TDMD (160,161). In addition, there are attempts that use *in vivo* CRISPR screening for the identification of miRNAs targets by reverting the effects of mutated miRNA targets by mutated seed sequences (162).

A main advantage of the reporter assays is the possibility to measure the effects of miRNA–target binding in a functional setting. There are, however, several drawbacks in part due to necessary transfections, which lead to non-physiological miRNA and/or 3′UTR-target concentrations. To address this problem, chemically inducible expression constructs have recently been tested in context with reporter assays, mimicking expression rates at physiological quantities (163). An additional drawback of the reporter assays is the common use of 3′UTR fragments instead of the full-length 3′UTRs. The potentially altered 3′UTR secondary structure of shorter fragments likely impacts the miRNA target binding. To address this caveat, efficient cloning protocols for the generation of full-length 3′UTR luciferase reporter constructs have recently been proposed (164,165). While a reporter-based analysis of 5’UTR reporter plasmids is also conceivable, it would be hard to implement the investigation of coding sequences. Furthermore, the assays are preferentially performed with cells that can readily be transfected with high efficiency like e.g. HeLa or HEK293 (Human Embryonic Kidney 293) cell lines (166,167). It is evident that their cellular context with the above-mentioned regulatory networks, including endogenous miRNA pools, ceRNAs and RBPs, is largely different from the cells of interest with the consequence that the miRNA target interaction detected in a reporter assay may not be found in other cell types. Finally, if done manually reporter assays are rather time consuming in that they require several steps including cloning of the 3′UTR sequence and transfection of both the recombinant and appropriate control constructs, i.e. empty reporter vectors and miRNA negative controls. Recently developed high-throughput miRNA interaction reporter (*HiTmIR*) assays can help to render these assays more efficiently (122,127).

In addition to reporter assays, MTIs are frequently analyzed by approaches that use Ago immunoprecipitation followed by an examination of the bound miRNA and mRNA fractions (157). There are various modifications of these approaches like ‘High-Throughput sequencing of RNA isolated by crosslinking immunoprecipitation’ (HITS-CLIP), ‘Cross-Linking Ligation and Sequencing of Hybrids’ (CLASH) and ‘Argonaute-RNA Immunoprecipitation’ (AGO-RIP). In HITS-CLIP assays, UV-irradiation induces crosslinking of cellular Ago proteins together with the incorporated miRNAs and the bound target mRNA fractions. After immunoprecipitation with Ago-specific antibodies, complexed RNA fractions are purified and subjected to sequencing analysis (168). In the CLASH assays, base paired miRNAs and mRNA target fractions are linked by a ligation step to form a hairpin-like structure. In this way, CLASH allows the mapping of specific miRNAs-target pairs with high precision (169,170). In the AGO-RIP assays, a native pulldown of Ago-RNA-complexes overcomes common inefficiency of cross-linking procedures (171,172). The newest precipitation-based technique includes the use of biotinylated miRNAs, a formalin cross-linking step and a streptavidin-based pull-down of miRNA–DNA-complexes for the detection of nuclear miRNA functions (173). A major advantage of the precipitation-based approaches is that many different cell types can be examined and not just very specific cell models, as is the case with reporter assays. This, however, not necessarily implies the absence of major limitations. Precipitation-based approaches usually require large amounts of the considered miRNA. High endogenous expression levels are commonly achieved by induced expression (172). Additionally, precipitation-based approaches are often done with highly reproductive cell lines to compensate for the overall material loss during multiple experimental steps (174,175). This of course entails all drawbacks associated with cultured cell lines (176) and renders conclusion on true *in vivo* binding effects of miRNA problematic. A rather elaborate methodology has recently been published describing an efficiency enhanced pulldown of Ago2 proteins using transgenic introduced HaloTags (177). Although this method allows the use of primary tissue material from transgenic animals, cell samples may still need to be pooled from different individuals to provide sufficient material for the subsequent sequencing analysis (177). This bears the risk of high cellular heterogeneity within the resulting bulk data. Another disadvantage is that precipitation-based techniques only detect miRNA–target binding without providing evidence for the functional relevance of such binding. Without this evidence, a detected binding may have also occurred by chance. This could explain why recently published CLIP data only poorly correlated with target repression data in human cell lines (178). Recent studies complemented Ago immunoprecipitation by reporter assays or by miRNA titration tests, thereby adding functional validation of the detected binding sites (179,180).

As addressed above, the measurement of expression levels of target mRNAs or proteins offers a straightforward readout for miRNA manipulations (147,148). This readout is, however, far from proving a link between a given miRNA and a biological effect. To robustly confirm such link in specific cellular contexts, appropriate assays are required. Although many targets like transcription factors offer themselves for specific downstream testing, frequent functional analyses in MTI studies address rather basic cellular functions e.g. by employing proliferation or viability assays without establishing a direct link to the actual miRNA target.

## Conclusion

Since the discovery of miRNAs, significant insights into the complexity of MTIs have been gained. There is an increasing understanding of the dynamics of miRNA regulations, which acknowledges the variability of miRNA binding constellations, the context dependent modulation of miRNA sequences, the alteration of miRNA subcellular localizations and the impact of various endogenous interaction partners. There is also an increasing awareness of the context dependency of MTIs. The continuous improvement of experimental strategies increasingly considers the cellular context of miRNA regulations thereby contributing to more reliable definitions of miRNAs and miRNA targets and in consequence to a holistic view of their roles in cellular regulatory networks.

## Data Availability

No new data were generated or analysed in support of this research.
